# Structural Features of Three Hetero-Galacturonans from *Passiflora foetida* Fruits and Their in Vitro Immunomodulatory Effects

**DOI:** 10.3390/polym12030615

**Published:** 2020-03-08

**Authors:** Ya Song, Peng Wen, Huili Hao, Minqian Zhu, Yuanming Sun, Yuxiao Zou, Teresa Requena, Riming Huang, Hong Wang

**Affiliations:** 1 Guangdong Provincial Key Laboratory of Food Quality and Safety, College of Food Science, South China Agricultural University, Guangzhou 510642, China; 2 Sericultural & Agri-Food Research Institute, Guangdong Academy of Agricultural Sciences; Key Laboratory of Functional Foods, Ministry of Agriculture; Guangdong Key Laboratory of Agricultural Products Processing, Guangzhou 510610, China; 3 Instituto de Investigacion en Ciencias de la Alimentacion CIAL (CSIC-UAM), Nicolas Cabrera, 9, Campus de Cantoblanco, Universidad Autonoma de Madrid, 28049 Madrid, Spain

**Keywords:** structural features, immunomodulatory effect, *Passiflora foetida* fruits, hetero-galacturonans

## Abstract

*Passiflora foetida* is a horticultural plant and vital traditional Chinese herbal medicine. In our previous study, the characterization and immuno-enhancing effect of fruits polysaccharide 1 (PFP1), a water-eluted hetero-mannan from wild *Passiflora foetida* fruits, were investigated. Herein, another three salt-eluted novel polysaccharides, namely PFP2, PFP3, and PFP4, were obtained and structurally characterized. The results showed that PFP2, PFP3, and PFP4 were three structurally similar hetero-galacturonans with different molecular weights of 6.11 × 10^4^, 4.37 × 10^4^_,_ and 3.48 × 10^5^ g/mol, respectively. All three of these hetero-galacturonans are mainly composed of galacturonic acid, galactose, arabinose (75.69%, 80.39%, and 74.30%, respectively), and other monosaccharides including mannose, fucose, glucose, ribose, xylose, and glucuronic acid (24.31%, 19.61, and 25.70%, respectively), although differences in their backbone structure exist. Additionally, immunomodulatory assay indicated that the three hetero-galacturonans possess the ability to promote the production of nitric oxide (NO), tumor necrosis factor-α (TNF-α), and interleukin-6 (IL-6) in RAW264.7 macrophages in a concentration-dependent manner (*p* < 0.05). Especially, PFP3 displayed a stronger enhancing effect than PFP2 and PFP4 at the minimum effective concentration. Therefore, the results suggested that the obtained three salt-eluted hetero-galacturonans, especially PFP3, could be utilized as immunomodulatory effectivity ingredients in nutritional/pharmaceutical industries.

## 1. Introduction

*Passiflora foetida*, known as “Long-zhu” in Chinese, belongs to the genus *Passiflora* and is mainly distributed in tropical or subtropical areas. Studies have reported that *P. foetida* fruits possessed a wide range of pharmacological activities, such as antibacterial, anti-osteoporotic, anti-inflammatory, and antioxidant properties [1,2,3,4]. In our previous study, a novel water-eluted hetero-mannan, namely PFP1, was separated from the crude *P. foetida* fruits polysaccharide (PFP). The hetero-mannan exhibited significant immuno-enhancing effect on RAW264.7 macrophages by promoting the production of NO and tumor necrosis factor-α (TNF-α) and interleukin-6 (IL-6). However, the obtained PFP1 was 10.3% of the crude *P. foetida* fruits polysaccharide, which means that the main components of PFP are not yet clear. Generally, the anion exchange matrix binds to negatively charged polysaccharide fractions, which could be eluted off by measures such as increasing the salt concentration in the eluent. Hence, further study on the salt-eluted PFP remains to be of great importance. Moreover, researches on the composition, structure and biological activity of these salt-eluted fractions from PFP are still scarce. 

Among the salt-eluted polysaccharides, such as galactans, glucans, galacturonans, galacturonans have attracted more attention in modern pharmacology [5,6,7]. It is one kind of polysaccharides with a backbone of galactopyranouronic (Gal*p*Ac) residues and mostly distributed in plants as heterozygotes, which have extensively gained concerns because of their multiple biological properties, especially the immunomodulatory activities [8,9,10,11,12]. Studies have shown that these galacturonans derived from different plants (such as sweet cherry, stem lettuce, *Gynostemma pentaphyllum* tea, and elderflowers) with different molecular weights (9~2490 kDa) and different percentages of galacturonic acid (17.4%–77.18%) exhibited various immunoregulatory effects on macrophages by enhancing production of NO and cytokines at the minimum effective concentration (0.8~100 µg/mL). Therefore, the structure-activity relationship between salt-eluted polysaccharides and its immunomodulatory activity need to be investigated.

In this study, PFP was further separated and eluted by anion exchange chromatography column (DEAE sepharose) with different and gradually increasing concentration of sodium chloride solution. Three novel polysaccharide fractions (PFP2, PFP3, and PFP4) were obtained and then structurally characterized with gel-permeation chromatography, connected with multi-angle light scattering and refractive index detectors (GPC-MALS-RI), Fourier transform-infrared spectroscopy (FT-IR), ion chromatography (IC), gas chromatography-mass spectrometry (GC-MS), methylation analysis, and nuclear magnetic resonance (NMR) spectroscopy. In addition, the immunomodulatory activity in vitro of PFP2, PFP3, and PFP4 on murine RAW264.7 macrophages was investigated. 

## 2. Materials and Methods

### 2.1. Plant Materials and Chemicals

Wild *P. foetida* fruits were collected from Dongfang city (Hainan, China). Standard monosaccharides (mannose, arabinose, fucose, glucose, ribose, galactose, xylose, glucuronic acid, and galacturonic acid), lipopolysaccharide (LPS), polymyxin B, trifluoroacetic acid (TFA) and acetonitrile (chromatographic grade) were the products of Sigma Chemical Co. (St. Louis, MO, USA). DEAE Sepharose fast flow and Sephadex G-100 were purchased from Whatman Ltd (London, UK). The macrophage RAW264.7 cell line was obtained from ATCC (Shanghai, China). Penicillin, streptomycin, fetal bovine serum (FBS) and Dulbecco’s Modified Eagle’s medium (DMEM) culture media were purchased from Gibco BRL (Gaithersburg, MD, USA). NO detection kit and ELISA kits (TNF-α and IL-6) for mice were purchased from Beyotime Institute of Biotechnology (Zhenjiang, Jiangsu, China). All other chemicals and reagents used in the experiments were an analytical grade (Yuanye Bio-Chem Technology Co. Ltd, Shanghai, China). 

### 2.2. Separation and Purification of Salt-Eluted Polysaccharides

The crude *P. foetida* fruits polysaccharide (PFP) was obtained via the same method as in our previous study [13]. Dried PFP was completely dissolved in deionized water (1:20, *w/v*), then loaded (50 mL) on a DEAE sepharose fast flow column (1.6 cm × 40 cm, Buchi, Flawil, Switzerland) and eluted subsequently with distilled water, 0.1, 0.2, and 0.3 mol/L NaCl solution at a flow rate of 1 mL/min. The total polysaccharide content of eluent (10 mL/tube) was analyzed by the phenol-sulfuric acid method [14]. The peaks were collected, lyophilized, and further purified on a Sephadex G-100 chromatography (1.6 cm × 60 cm, Buchi, Flawil, Switzerland) eluted with 0.1 mol/L NaCl solution at a flow rate of 0.2 mL/min. The purified polysaccharide fractions were obtained after the concentration and lyophilization.

### 2.3. UV-Visible and FT-IR Spectra Analysis

A NanoDrop 2000c UV-Visible spectra (Thermo Fisher Scientific, Waltham, MA, USA) was utilized to check the presence of a nucleic acid-protein of purified polysaccharides solution (50 µg/mL) in the range of 190 to 800 nm. The FT-IR spectra of these samples (KBr pellets) were obtained via a VERTEX 33 FT-IR instrument (Bruker, Rheinstetten, Germany) in the frequency range from 4000 to 400 cm^−1^.

### 2.4. Molecular Weight Analysis

The molecular weight distribution of these samples was determined by GPC on a Waters Series 1500 system, equipped with a TSKgel G3000PWXL column (7.8 mm × 300 mm, Tosoh, Japan) and a TSKgel G6000PWXL column (7.8 mm × 300 mm, Tosoh, Japan). The system was connected with a Wyatt MALS detector (DAWN HELEOS Ⅱ, Wyatt technology, Santa Barbara, CA, USA) and a Wyatt RI detector (Optilab Trex, Wyatt technology, Santa Barbara, CA, USA). Each polysaccharide fraction (10 ± 0.05 mg) was added to 90% dimethylsulfoxide (DMSO, *v/v*, 1 mL) overnight at 100 °C, followed by centrifugation. Then, the precipitate was washed twice with anhydrous ethanol (3 mL) to remove the residual DMSO. Afterwards, the dried precipitate was dissolved in 0.1 mol/L NaNO_3_ (3 mL) at 121 °C for 20 min, and centrifuged (12,000 rpm, 10 min) for analysis. The sample (100 µL) was eluted off by NaNO_3_ solution (0.1 mol/L) at a flow rate of 0.4 mL/min and the column oven was kept at 60 °C. The data was analyzed using ASTRA6.1 software (Wyatt Technology Corporation, Santa Barbara, CA, USA).

### 2.5. Monosaccharide Composition Analysis 

Each polysaccharide fraction (5 ± 0.05 mg) was hydrolyzed by using 4 mL TFA (2 mol/L) at 121 °C for 2 h in a sealed tube, which was repeated three times. The hydrolytic product was extracted by ethanol and dried by a rotary concentrator till the excess TFA was completely removed, then dissolved in ultrapure water (2 mL) and filtered (0.22 µm). Whereafter, the hydrolyzed and filtered samples (25 µL) were loaded on the Thermo Fisher IC system (ICS5000, Waltham, MA, USA) with an efficient anion exchange column of DionexTM CarbopacTM PA20 column (150 mm × 3 mm) and a Dionex pulsed amperometric detector equipped with an Au electrode (Thermo Fisher Scientific). A (deionized water), B (NaOH, 0.2 mol/L) and C (NaOH, 0.2 mol/L; NaAC, 0.5 mol/L) were used as the mobile phases. The gradient was as follows: 0–25 min (97.5%A and 2.5%B), 25.1–40 min (77.5% A, 2.5% B, and 20% C), 40.1–50 min (100%C), 50.1–60 min (97.5% A and 2.5% B). The flow rate was 0.5 mL/min. Mannose, arabinose, fucose, glucose, ribose, galactose, xylose, glucuronic acid, and galacturonic acid were used as standards. Instrument control and peak evaluation were done with Chromeleon 7.2 (Thermo Fischer Scientific).

### 2.6. Molecular Morphological analysis

Dried polysaccharide samples were flimsily plated with platinum and visualized through an Zeiss SEM system (LEO1530VP, Oberkochen, Germany) at a 15 kV accelerating voltage with different image magnifications of 1000×, 3000×, and 5000×, respectively.

### 2.7. Methylation Analysis

The reduction of uronic acids and methylation analysis were carried out as described previously [15,16] with minor modification. Briefly, polysaccharide samples (10 ± 0.05 mg) were reduced with sodium borohydride (NaBH_4_) and boron sodium deuteride (NaBD_4_), then dialyzed and lyophilized to acquire the reduzates. Then the reduzates were methylated with DMSO/NaOH and CH_3_I. The FT-IR spectrum was utilized to confirm that the methylations were completed.

The permethylated samples were successively hydrolyzed with 1 mL of 2 M TFA at 121 °C for 1.5 h, then reduced with NaBD_4_ and acetylated with acetic anhydride. The eventual derivatives were dissolved in chloroform and injected into a GC-MS analysis on an Agilent 6890A-5975C instrument equipped with Agilent HP-5 capillary column (Santa Clara, CA, USA, 19091S-433, 30.0 m × 0.25 mm × 0.25 μm). The column temperature was set to 140 °C during injection, and held for 2 min, then increased by 3 °C/min to 230 °C and maintained at 230 °C for 3 min. Helium was used as the carrier gas. Mass spectra were interpreted to identify the compounds that corresponded to each peak. The molar ratio of each residue was calculated based on peak areas.

### 2.8. NMR Analysis 

The linkages and configuration of these purified polysaccharides were identified using ^1^H and ^13^C NMR spectroscopy. Each polysaccharide fraction (20 ± 0.05 mg) was fully dispersed in ultrapure water (1 mL), centrifuged to remove the precipitation, and the supernatant was lyophilized. The operation was repeated three times, and then the finally lyophilized samples were completely dispersed in 0.5 mL D_2_O, including 1 µL acetone [17]. NMR was recorded on a Bruker AVANCE 600 MHz spectrometer (Billerica, MA, USA). The spectra were observed at 25 °C from 4000 scans. 

### 2.9. Immunomodulatory Effect Analysis

RAW264.7 murine macrophage cells (500 µL) were seeded into a 24-well microplate at a density of 1 × 10^5^ cells/mL and cultured in DEME culture media (supplemented with 10% (*v/v*) FBS and 1% (*v/v*) penicillin-streptomycin) at 37 °C in 5% CO_2_ humidified atmosphere. After cell adhesion had formed or happened for 24 h, the conditioned culture mediums were replaced with 1 mL new DEME culture medium, in which containing different concentrations of PFP2, PFP3, and PFP4 (0.032, 0.16, 0.8, 4, or 20 µg/mL) or culture medium (negative control) for 24 h. In addition, cells cultured in LPS (1 µg/mL) were used as positive control. To exclude the probability of LPS contamination during the polysaccharide isolation period, each polysaccharide fraction sample (20 µg/mL) was blended with various concentrations of polymyxin B (0, 100, or 200 µg/mL) for 0.5 h before added to the medium [10]. The conditioned culture medium was collected and centrifuged (12,000 rpm, 10 min, 4 °C) to determine the content of NO, TNF-α and IL-6 from RAW264.7 cells by using commercial kits in accordance with the manufacturer’s instructions. The level of NO in the supernatant was measured using Griess method and was estimated from a sodium nitrite standard curve. Cytokines (TNF-α and IL-6) in the supernatant were measured by ELISA assay according to manufactures’ instructions. The absorbance was determined at 540 nm using a microplate reader.

### 2.10. Statistical Analysis

All results were presented as the mean ± standard error of the mean (SEM), and data were analyzed using a one-way analysis of variance. *P* values less than 0.05 were considered statistically significant.

## 3. Results and Discussion

### 3.1. Extraction and Purification of the Polysaccharide Fractions

**Figure 1:**
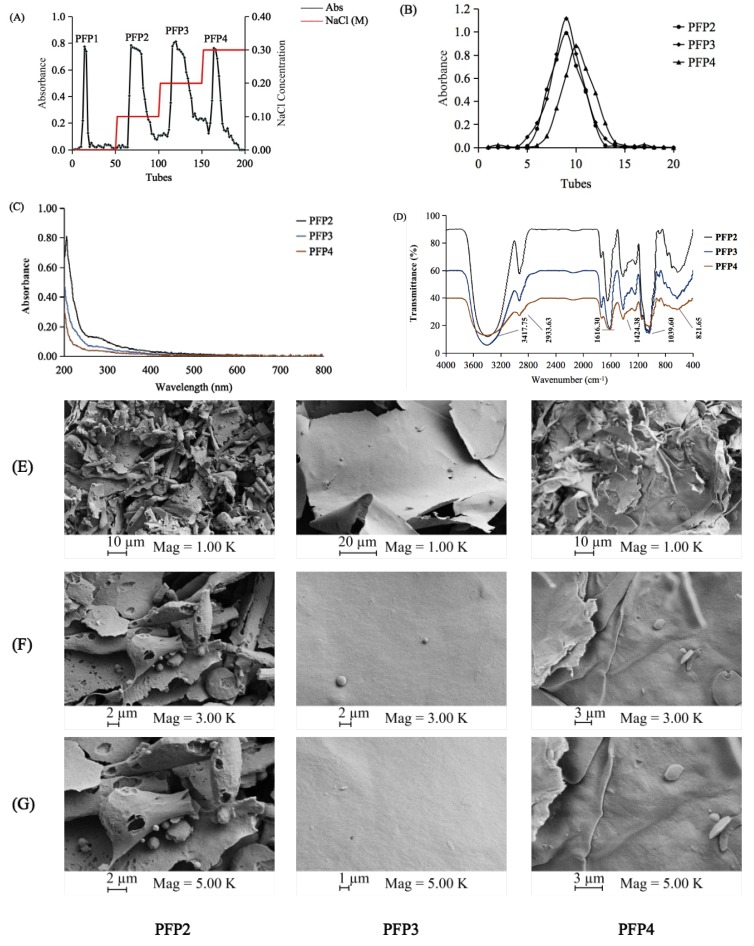
Separation and spectra of PFP2, PFP3, and PFP4. DEAE-cellulose elution curve (**A**); Sephadex G-100 elution curve (**B**); The UV–Vis spectra (**C**); IR spectra (**D**); and SEM images (**E**: ×1k; **F**: ×3k; **G**: ×5k).

The PFP was separated on a DEAE-cellulose Fast Flow column, and a separation flow diagram for PFP was illustrated in Figure 1A. In addition to PFP1, the other three fractions, namely PFP2, PFP3, and PFP4, were obtained according to the order of the elution peaks with the yields of 19.8%, 26.2%, and 12.7%, respectively. These three salt-eluted fractions were further purified by a Sephadex G-100 column. Three single and symmetrically sharp peaks were presented in Figure 1B. Results revealed that PFP2, PFP3, and PFP4 were homogeneous polysaccharides. After purification, PFP2, PFP3, and PFP4, with the total carbohydrate purity of 80.1%, 76.3%, and 78.2%, respectively, were subjected to further chemical analysis.

### 3.2. Analysis of UV–Vis and FT-IR Spectra 

UV–Vis spectra (Figure 1C) showed that PFP2, PFP3, and PFP4 had almost no visual absorption at 260 or 280 nm, suggesting the deficiency of protein or nucleic acids (< 3%) in these polysaccharides. 

Like PFP1, the FT-IR spectra of the PFP2, PFP3, and PFP4 fractions showed representative absorbing bands of acidic polysaccharides in the range of 4000–400 cm^−1^ (Figure 1D). The vibration regions of PFP2, PFP3, and PFP4 approximately at 3417.75 cm^−1^ may be due to the stretching vibration of O–H. The broad bands at 2933.63 cm^−1^ were assigned to the stretching vibration of C-H in the sugar ring [18]. Stretching peaks at 1616.30 cm^−1^ represented the specific stretching vibration of C=C bonds, as well as at 1424.38 cm^−1^, which could be attributed to symmetric COO–, representing the presence of uronic acids [19]. The strong peaks observed at 1000–1200 cm^−1^ (1039.60 cm^−1^) were assigned to their C–O–H and C–O–C stretching vibrations. The weak absorption bands at around 821.65 cm^−1^ might be combined with a-pyranose [20]. These results further confirmed that PFP2, PFP3, and PFP4 were acidic polysaccharides.

### 3.3. Morphological Properties

The morphological properties of PFP2, PFP3, and PFP4 through SEM (Figure 1E–G) were performed at magnifications of 1000×, 3000×, and 5000×. These three polysaccharides were principally formed of various anomalous flakes with increasing degrees of stretch, which might due to the discrepant highly branching construction [21]. Differently, PFP2 had a rough laminated structure with some small distinct pore, while PFP3 and PFP4 had a flake-like structure with a smooth surface. The diversity might be caused by the differences in the water holding capacity and the removal of water during the lyophilization process [22]. 

### 3.4. Molecular Weight and Monosaccharide Composition

As shown in Table 1, the molecular weights of PFP2, PFP3, and PFP4 were 6.11 × 10^4^ g/mol, 4.37 × 10^4^ g/mol, and 3.48 × 10^5^ g/mol, respectively. Polydispersity index (M_w_/M_n_) of PFP2 (1.58), PFP3 (3.82), and PFP4 (6.18) were lower than that of PFP1 (7.14), which suggested that these three fractions were polydisperse polysaccharides with a lower distribution of molecular weights than that of PFP1 [13]. Combined with the molecular weight of *P. edulis* polysaccharide (6.0 × 10^4^ g/mol) [23], we can see that the polysaccharides extracted from *Passiflora* spp. fruits possessed relatively high molecular weight. Among these polysaccharide fractions, PFP3 had the lowest molecular weight, and PFP4 had the highest molecular weight. 

**Figure 2:**
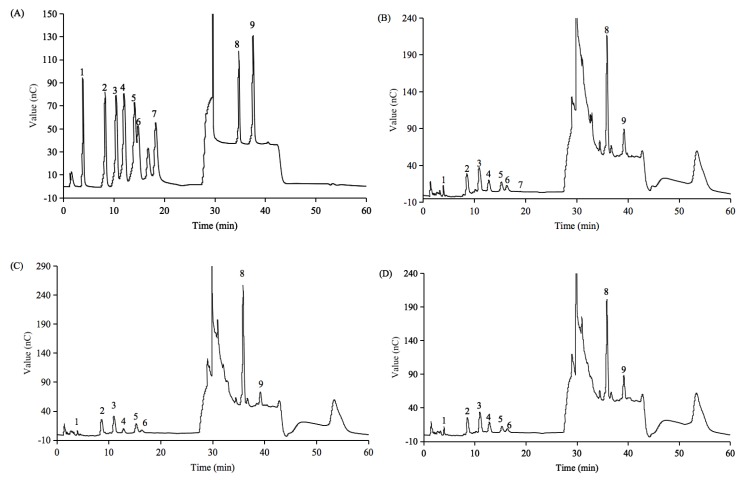
The ion chromatography (IC) chromatograms of standard monosaccharides. 1, fucose; 2, arabinose; 3, galactose; 4, glucose, 5, xylose; 6, mannose; 7, ribose; 8, galacturonic acid; and 9, glucuronic acid (**A**); The IC chromatograms of PFP2 (**B**), PFP3 (**C**), and PFP4 (**D**).

As illustrated in Figure 2, nine monosaccharide compositions were determined by an ion chromatograph. PFP2, PFP3, and PFP4 contained two uronic acids (glucuronic acid and galacturonic acid) and six neutral monosaccharides (mannose, arabinose, fucose, glucose, galactose, and xylose). Traces of ribose was detected only in PFP2. In addition, galacturonic acid, galactose, and arabinose were the main sugar units of these acidic polysaccharides (Table 1), this was completely different from PFP1, which was mainly composed of mannose and galactose. Similar results were observed in *P. edulis* polysaccharide (PFCM), which was dominated by galacturonic acid (44.2%) [23]. The results shown above speculated that PFP2, PFP3, and PFP4 might have linear galacturonans and heterogeneous sugars branched structures.

### 3.5. Methylation Analysis 

More structural information of PFP2, PFP3, and PFP4 were measured by methylation analysis. As shown in Table 2, GC-MS results indicated that PFP2, PFP3, and PFP4 have eight same types of sugar linkages (1-linked arabinofuranose (Ara*f*), 1-linked xylopyranose (Xyl*p*), 1-linked galactopyranose (Gal*p*), 1, 4-linked Gal*p*Ac, 1, 4-linked mannopyranose (Man*p*), 1, 3-linked Gal*p*, 1, 3, 4-linked Gal*p*Ac, 1, 3, 6-linked Gal*p*) at different molar percentage ratios, respectively. These results showed that the most common derivative of PFP2, PFP3, and PFP4 are Gal*p* residues (57.63%, 34.61%, and 43.04%, respectively) and Gal*p*Ac residues (22.65%, 43.18%, and 23.18%, respectively), which were virtually in accordance with the general monosaccharide composition, but different from those of PFP1 that was dominated by Man*p* residues. The existence of 2, 3, 4, 6-Me_4_-Gal*p* derivatives demonstrated that a portion of Gal*p* was participated in the structure of PFP2, PFP3, and PFP4 as non-reducing terminal units. Ara*f* and Xyl*p* non-reducing terminals were also appeared, on account of the existence of 2, 3, 5-Me_3_-Ara*f* and 2, 3, 5-Me_3_-Xyl*p* derivatives. The detection of large amounts of 2, 3, 6-Me_3_-Gal*p*Ac, and 2, 6-Me_2_-Gal*p*Ac in the eventual derivatives demonstrated that the backbone of PFP2, PFP3, and PFP4 mainly composed of (1→4)- and (1→3,4)-Gal*p*Ac residues. The presence of 2, 4, 6-Me_3_-Gal*p,* and 2, 4-Me_2_-Gal*p* derivatives indicated the participation of Gal*p* units in these polysaccharides structure as (1→3)- and (1→3,6)-Gal*p* residues. In addition, Man*p* derivatives, presented in PFP1, were discovered in the form of 2, 3, 6-Me_3_-Man*p* products in PFP2, PFP3, and PFP4, suggesting their linkage through (1→4)-Man*p*.

The presence of 2, 3, 4-Me_3_-Glc*p* and 2, 3, 4-Me_3_-Gal*p* indicating the linkages of (1→6)-Glc*p* and (1→6)-Gal*p* both in PFP2 and PFP4. Besides, the signals of 3, 4, 6-Me_3_-Gal*p,* and 4, 6-Me_2_-Gal*p*, suggested the existence of a (1→2)-Gal*p* in PFP3 and a (1→2,3)-Gal*p* in PFP2, respectively. In addition to the differences in molar ratios of common residues, the presence or absence of these residues may be the cause of structural differences among PFP2, PFP3, and PFP4. 

### 3.6. NMR Analysis

**Figure 3:**
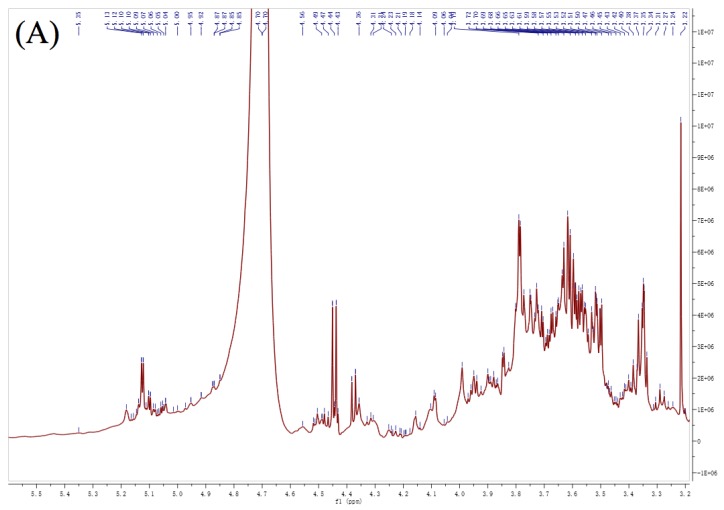

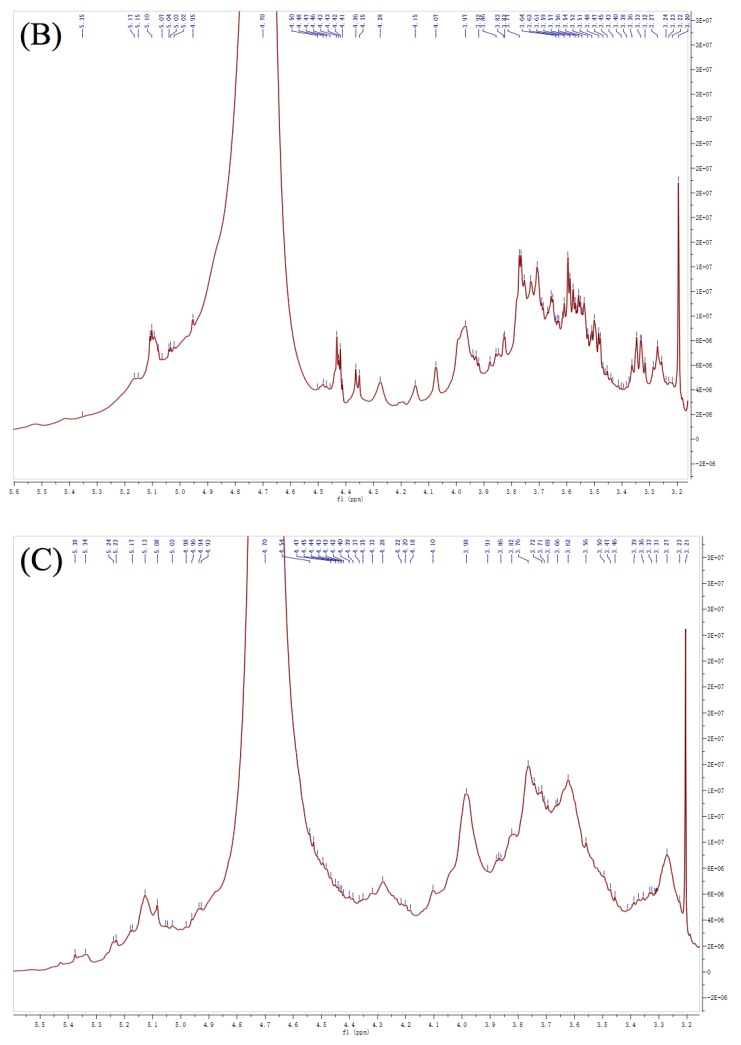

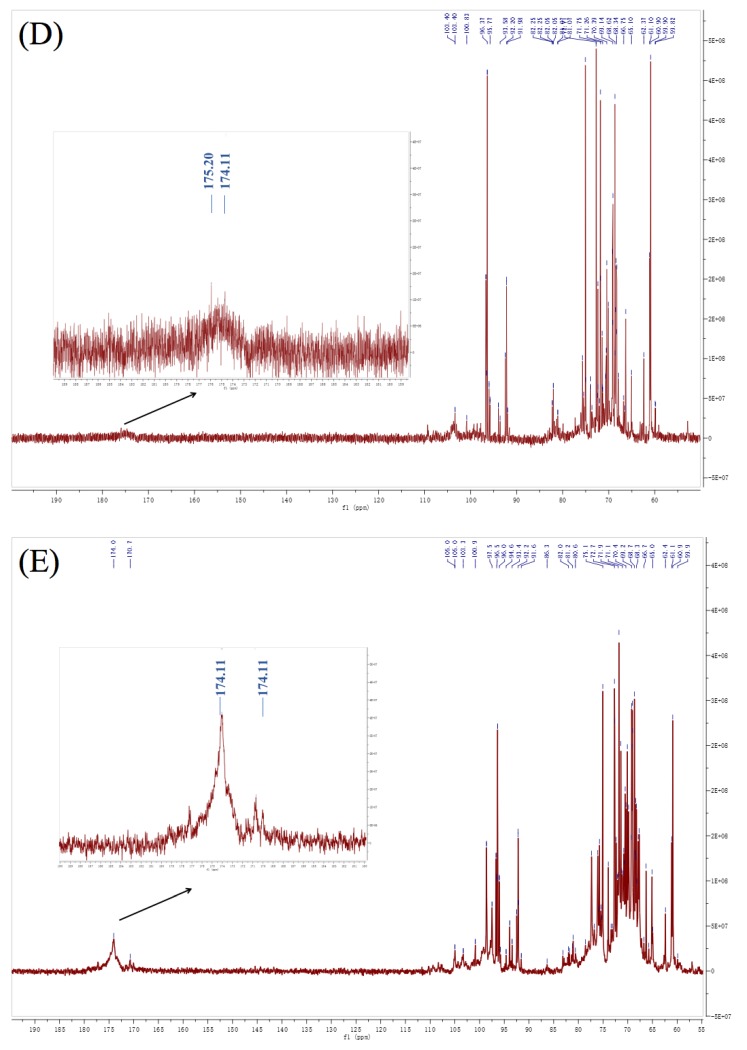

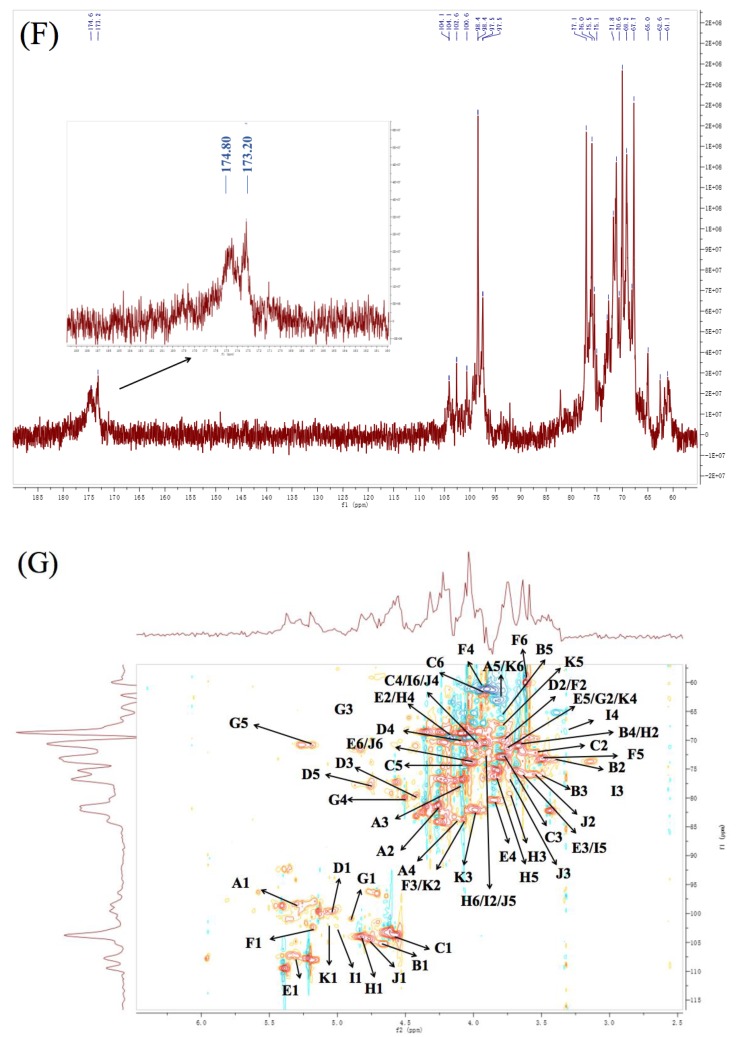

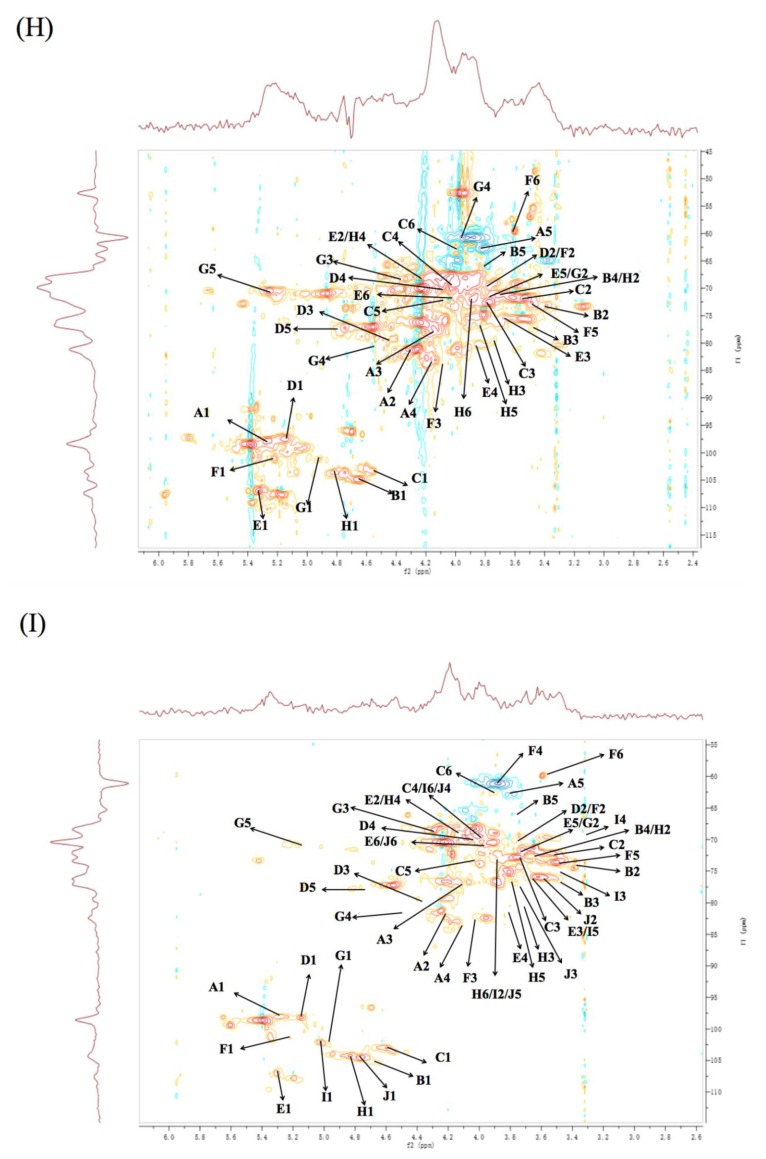

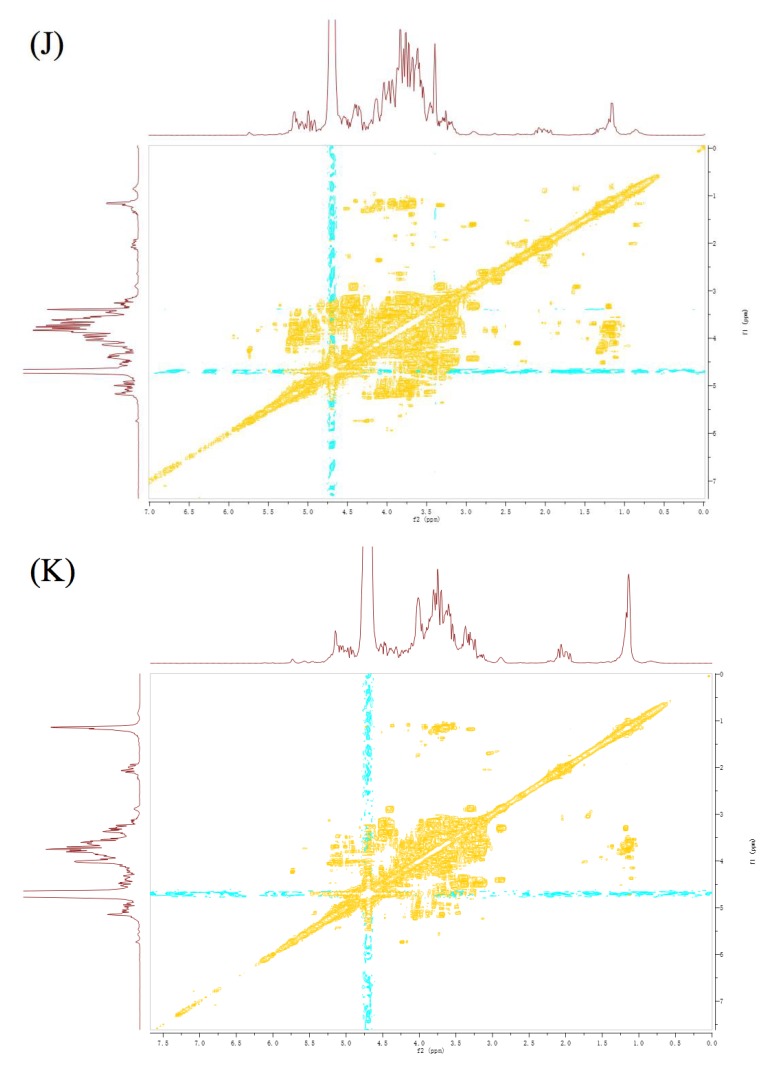

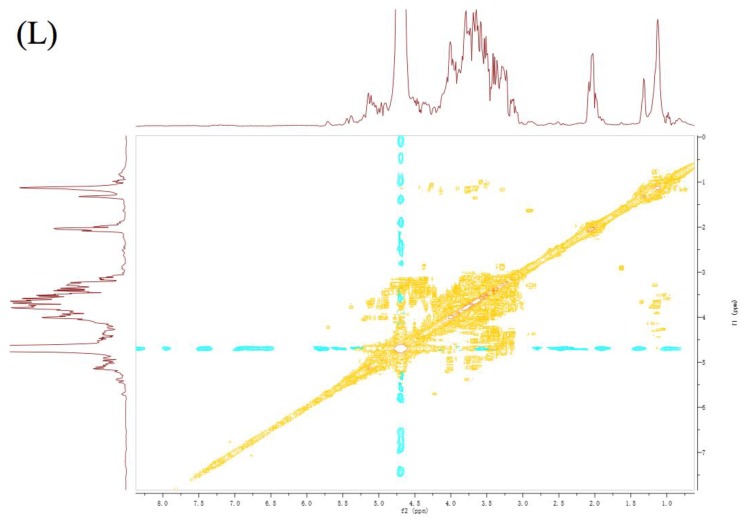
The NMR spectra of PFP2, PFP3, and PFP4 in D_2_O. ^1^H spectra (**A**–**C**); ^13^C spectra (**D**–**F**); Heteronuclear single quantum coherence (HSQC) spectra (**G**–**I**); ^1^H-^1^H Correlation spectroscopy (COSY) spectra (**J**–**L**).

As illustrated in Figure 3, the ^1^H- and ^13^C-NMR spectra of these three polysaccharides had similar signal patterns. PFP2, PFP3, and PFP4 had eight similar anomeric proton signals at δ 5.30/5.27/5.28, 5.39/5.36/5.35, 4.57/4.56/4.58, 5.07/5.02/5.14, 5.33/5.32/5.30, 5.14/5.22/5.21, 4.83/4.92/4.97 and 4.90/4.81/4.82, which were ticketed **A**, **B**, **C**, **D**, **E**, **F, G,** and **H**, respectively. Besides, both PFP2 and PFP4 had two chemical shift signals at δ 5.00/5.01, 4.76/4.74, which were assigned **I** and **J**, respectively. PFP2 had a unique residue, appointed as **K**, in the anomeric proton signals at δ 5.07. Signals of 1, 2-Gal*p* residue were not detected due to low resolution. Signals within the range of 3.32−5.39 ppm (^1^H NMR) and 59.55−175.20 ppm (^13^C NMR) represented a typical characteristic of polysaccharides. All the ^1^H and ^13^C (Table 3) were portioned as thoroughly as possible according to 2D NMR analysis and on literature values [6,10,24,25,26,27,28,29].

Generally, the anomeric signals represent the type of *α*-configuration in 5.0–5.8 ppm region and represent the *β*-configuration in 4.4–5.0 ppm region. Thus, residues **A**, **D**, **E**, **F**, **I,** and **K** were *α*-configuration and residues **B**, **C**, **G**, **H,** and **J** were *β*-configuration. According to the methylation analysis, Gal*p*Ac residues were expected to be the most abundant residues. The residues **D** and **G** had anomeric signals at δ 5.07/5.02/5.14 and 4.83/4.92/4.97 ppm, while signals at δ 175.2/174.0/173.2 and 103.99/100.96/102.64 ppm in the ^13^C NMR indicating that the residues **D** and **G** might be galacturonic acid moiety. Based on the proton chemical shifts, ^1^H and ^13^C chemical shifts indicated by ^1^H-^1^H correlation spectroscopy (COSY) and heteronuclear single quantum coherence (HSQC) spectra (Figure 3), the chemical shifts of residues **D** and **G** consistent nearly with the reported values for a methyl glycoside of 1, 4-linked Gal*p*Ac and 1, 3, 4-linked Gal*p*Ac. In consideration of the results of FT-IR spectra, methylation analysis and NMR results, it could be deduced that the backbones of PFP2, PFP3, and PFP4 were mainly composed of (1→4)-linked-Gal*p*Ac and (1→3, 4)-linked-Gal*p*Ac residues [6,10,26,30].

Considering the results of methylation analysis, the present anomeric signals indicated the presence of three same galactopyranose residues in repetitive units of PFP2, PFP3, and PFP4. The chemical shift signals at δ 103.73/103.31/103.14 (4.57/4.56/4.58), 99.55/100.96/101.54 (5.14/5.22/5.21), and 100.91/103.58/104.12 (4.90/4.81/4.82) ppm were appointed to the C-1(H-1) of T-linked Gal*p* residues (residues **C**), (1→3)-linked Gal*p* residues (residues **F**) and (1→3,6)-linked Gal*p* residues (residues **H**), respectively [27]. Furthermore, the signals of residues **J** showed their anomeric chemical shifts at 4.76/4.76 ppm and 104.35/104.59 ppm, respectively, in PFP2 and PFP4, which were well matched with the methylation analysis results of PFP2 and PFP4 as shown in Table 2 and were attributed to the C-6 of T-linked Gal*p* [31]. For residues **K**, the unique glycosidic linkage pattern in PFP2, their signals of C-1, C-2, C-3, C-4, C-5, and C-6 were observed at δ 101.85, 83.61, 82.13, 71.33, 68.05, and 63.09 ppm revealed the presence of (1→2,3)-linked Gal*p* residues [29]. 

The chemical shifts in ^1^H-NMR of PFP2, PFP3, and PFP4 in the anomeric proton of residues **A** were δ 5.30, 5.27, and 5.28 ppm, respectively, while the matching chemical shifts in ^13^C NMR of the anomeric carbon were δ 98.32, 97.82, and 98.26 ppm, respectively. Above all of these results are in accordance with the FT-IR and methylation results, demonstrating that residues **A** were T-linked Ara*f* [27]. Three signals (residues **B**) showed up at 4.67, 4.66, and 4.64 ppm, which were assigned to anomeric protons of T-linked Xyl*p* residues of PFP2, PFP3, and PFP4, respectively [25]. All the ^1^ H and ^13^ C chemical shifts of residue **E** assigned by COSY and HSQC in this study were consistent with previous studies [28], and the downfield shift of C-4 (80.87, 80.13, and 80.89 ppm) carbon signals with respect to standard values of methyl glycosides indicated that residues **E** were (1→4)-linked Man*p* residues. Meanwhile, (1→6)-linked Glc*p* (residues **I**) showed their anomeric chemical shifts at 4.96/4.94 ppm and 104.01/104.05 ppm, respectively, for ^1^H and ^13^C, which were well matched with the methylation analysis results of PFP2 and PFP4 as shown in Table 2 [27].

Therefore, considering all the information obtained from NMR spectra, methylation analyses and literature comparisons, PFP2, PFP3, and PFP4 were proven to have a linear homogalacturonan (HG) and →3, 4)-*β*-_D_-Gal*p*Ac-(1→, with branches at the *O*-4 position, consisting of Ara*f,* Xyl*p*, Man*p* and Gal*p* residues, including *α*-_L_-Ara*f*, *β*-_D_-Xyl*p*, *β*-_D_-Gal*p*, →4)-*α*-_L_-Man*p*-(1→, →3)-*α*-_L_-Gal*p*-(1→ and →3, 6)-*β*-_D_-Gal*p*-(1→. Furthermore, both PFP2 and PFP4 had the side chains of →6)-*β*-_D_-Gal*p*-(1→ and →6)-*α*-_L_-Glc*p*-(1→, while only PFP2 has a branch of →2, 3)-*α*-_L_-Gal*p*-(1→. The structures of these three hetero-galacturonans were completely different from PFCM, which has linear homogalacturonan (HG) and neutral sugar-branched rhamnogalacturonan-1 (RG-1) structures [23].

### 3.7. Immunomodulatory Effect

**Figure 4:**
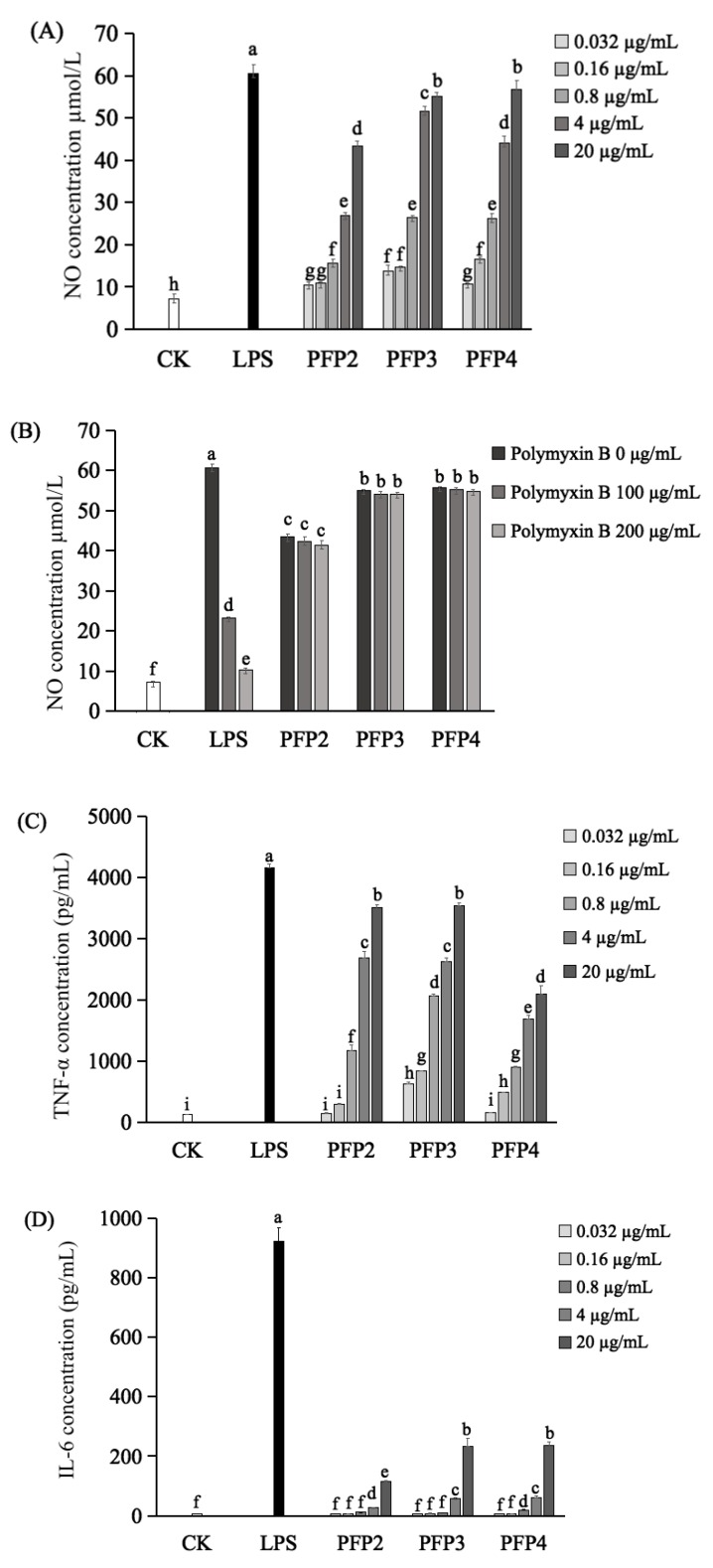
NO release detection of RAW264.7 cell activation. By LPS, PFP2, PFP3, and PFP4 (**A**). By LPS, PFP2, PFP3, and PFP4 with polymyxin B (**B**). Elisa detection of tumor necrosis factor-α (TNF-α) (**C**) and interleukin-6 (IL-6) (**D**). Data were presented as mean ± SD. Different lowercase alphabet letters (a–i) were significantly different if *p* < 0.05.

Macrophage activation is known as a primary and necessary step for innate immune system stimulation. The cytokines released from activated macrophages, such as NO, TNF-α and IL-6, are important in immune responses [11]. Thus, the immunomodulatory activity of these three hetero-galacturonans on macrophage cells RAW264.7 was studied in vitro. For the release of NO, the results showed that PFP2, PFP3, and PFP4 remarkably promoted the secretion of NO from RAW264.7 cells with significant dosage effects (*p* < 0.05) in the concentrations of 0.032 to 20 µg/mL. (Figure 4A). Especially, compared to PFP2 and PFP4, PFP3 possessed better ability to increase NO production at the minimum effective concentration of 0.032 µg/mL (*p* < 0.05). Under the action of treated maximum concentration (20 µg/mL) of PFP2, PFP3, and PFP4, the NO productions were 43.363 µmol/L, 55.040 µmol/L, and 56.748 µmol/L, respectively, which were significantly lower than that of LPS-treated group (60.608 µmol/L) at the concentration of 1 µg/mL (*p* < 0.05). Since the overproduction of NO could cause apoptosis in macrophages, the above results suggested that the macrophage activation of these three hetero-galacturonans were more moderate than that of LPS. Meanwhile, it can be seen that the activities of PFP2, PFP3, and PFP4 could not be neutralized by polymyxin B, indicating that they were free of LPS (Figure 4B). 

Regarding the secretion of TNF-α and IL-6 in RAW264.7 macrophages, ELISA results indicated that the cellular release of TNF-α (Figure 4C) was increased by PFP2, PFP3, and PFP4, with a remarkable concentration-dependent manner at the concentrations of 0.032 to 20 µg/mL. Additionally, the PFP3 displayed a stronger ability to enhance the secretion of TNF-α than PFP2 and PFP4 at the minimum effective concentration of 0.032 µg/mL (*p* < 0.05). Meanwhile, all these three hetero-galacturonans could promote the secretion of IL-6 (Figure 4D) with a significant concentration-dependent manner at the concentrations of 4 and 20 µg/mL (*p* < 0.05). These results of the present study were in agreement with the studies on hetero-galacturonans obtained from sweet cherry and stem lettuce [10,11]. Therefore, these results suggested that PFP2, PFP3, and PFP4 may play an immunomodulatory role through promoting the secretion of TNF-α, IL-6, and NO in RAW264.7 macrophages, which are essential for killing pathogens, microorganisms, and coordinating various biological activities as intracellular messenger molecules [5]. 

Immunomodulatory activity is one of the most important activities of galacturonans, but the structure–function relationship of galacturonans is still not clear. In the previous studies, several hetero-galacturonans obtained from sweet cherry [10], stem lettuce [11], *Tanacetum vulgare* [32], and elderflowers [33] that consisted of (1→4)-Gal*p*Ac backbone remarkably promoted the production of NO and cytokines from macrophages in a dose-dependent manner at concentrations between 25 and 200 µg/mL. It can be inferred the main linkage-type of polysaccharides, especially the (1→4)-Gal*p*Ac may be responsible for the macrophages activation. However, the main linkage type is not the only determinant of the immunomodulatory effect of polysaccharides. In this study, the immunomodulatory activity of PFP3 was significantly higher than that of the other two fractions. This phenomenon was in accordance with the study on lettuce polysaccharides (SLP-1 and SLP-2), which reported that SLP-2, with lower molecular weight (44 kDa) and the higher galacturonic acid content (69.5%), had higher macrophages activation effect than SLP-1, with the higher molecular weight (90 kDa) and the lower galacturonic acid content (17.6%) [11]. Taken together, it could be speculated that the relative high galacturonic acid content as well as the relatively low molecular weight of galacturonans may be responsible for the higher immunomodulatory effect. Therefore, although the structure-function relationship of galacturonans is still not explicit, the similarity of structural features like PFP2, PFP3, and PFP4 might be a part of the reasons for their immunomodulatory effect.

## 4. Conclusions

In conclusion, three novel salt-eluted polysaccharides, namely PFP2, PFP3, and PFP4, were obtained from the fruits of *P*. *foetida*. The structural analysis revealed that PFP2, PFP3, and PFP4 were three hetero-galacturonans with different molecular weights and composed of the same monosaccharide units but with different molar ratios. PFP2, PFP3, and PFP4 had similar glycosidic linkage patterns, which were proven to have a linear homogalacturonan (HG) and →3, 4)-*β*-_D_-Gal*p*Ac-(1→, with branches at the *O*-4 position, consisting of Ara*f,* Xyl*p*, Man*p,* and Gal*p* residues in different molar ratios. In addition, PFP2, PFP3, and PFP4 showed significant immunomodulatory effect, which similar to most previously reported plant galacturonans. These results indicated that PFP2, PFP3, and PFP4 have the potential to be novel immunomodulators for use in pharmaceuticals or functional foods and are worth further research.

## Figures and Tables

**Table 1:** Molecular, conformation parameters and monosaccharide compositions (%) of PFP2, PFP3, and PFP4.

Parameters	PFP2	PFP3	PFP4
Molecular and conformation parameters			
M_n_ (g/mol)	(3.86 ± 0.01) × 10^4^	(1.15 ± 0.02) ×10^4^	(5.63 ± 0.01) ×10^4^
M_w_ (g/mol)	(6.11 ± 0.01) × 10^4^	(4.37 ± 0.01) ×10^4^	(3.48 ± 0.01) ×10^5^
M_w_/M_n_	1.58 ± 0.01	3.82 (±0.02)	6.18 (±0.01)
Monosaccharide compositions ratio (%)			
Fucose	1.61	0.99	1.82
Arabinose	17.94	14.68	15.78
Galactose	26.99	18.37	21.56
Glucose	5.75	2.81	7.37
Xylose	5.62	9.14	4.61
Mannose	5.99	3.15	4.75
Ribose	0.02	NA^a^	NA^a^
Galacturonic acid	30.76	47.34	36.96
Glucuronic acid	5.32	3.52	7.15

^a^ NA, not detected.

**Table 2:** Methylate analysis data of PFP2, PFP3, and PFP4.

Retention Time (min)	Methylated Sugars	Type of Linkage	Molar Ratios (%)			Mass Fragments
			PFP2	PFP3	PFP4	
8.136	2, 3, 5-Me_3_-Ara*f*	T-Ara*f*	1.46	2.21	3.32	43, 71, 88, 101, 118, 129, 161, 191
9.164	2, 3, 5-Me_3_-Xyl*p*	T-Xyl*p*	8.03	16.45	13.83	43, 71, 87, 101, 102, 118, 129, 146, 162
13.675	2, 3, 4, 6-Me_4_-Gal*p*	T-Gal*p*	16.42	20.37	15.32	43, 71, 87, 102, 118, 129, 145, 161, 205
15.544	3, 4, 6-Me_3_-Galp	1, 2-Gal*p*	ND	7.11	ND	43, 71, 88, 100, 129, 145, 161, 190, 205
15.927	2, 3, 6-Me_3_-Gal*p*Ac	1, 4-Gal*p*Ac	12.96	30.21	15.69	43, 75, 99, 117, 159, 203, 233, 301, 318
16.143	2, 3, 6-Me_3_-Man*p*	1, 4-Man*p*	7.71	3.54	10.05	43, 71, 87, 102, 118, 162, 203, 233, 277
16.468	2, 4, 6-Me_3_-Gal*p*	1, 3-Gal*p*	15.46	5.36	12.77	43, 71, 87, 101, 118, 129, 161, 234, 277
16.635	2, 3, 4-Me_3_-Glc*p*	1, 6-Glc*p*	2.51	ND	6.58	43, 71, 87, 99, 102, 118, 129, 162, 189
17.607	2, 3, 4-Me_3_-Gal*p*	1, 6-Gal*p*	8.99	ND	6.91	43, 71, 87, 99, 102, 118, 129, 189, 233, 271
18.045	2, 6-Me_2_-Gal*p*Ac	1, 3, 4-Gal*p*Ac	9.69	12.98	7.49	43, 87, 118, 129, 161, 262, 305
18.104	4, 6-Me_2_-Gal*p*	1, 2, 3-Gal*p*	1.07	ND	ND	43, 86, 101, 129, 161, 202, 262
20.47	2, 4-Me_2_-Gal*p*	1, 3, 6-Gal*p*	15.70	1.77	8.04	43, 87, 101, 118, 129, 189, 234, 305

^a^ ND, not detected.

**Table 3:** ^1^H and ^13^C NMR Spectral assignments for PFP2, PFP3, and PFP4.

Residues	Linkage			1	2	3	4	5	6
**A**	*α*-_L_-Ara*f*	PFP2	H	5.39	4.26	4.09	4.14	3.81	
			C	109.45	81.21	77.67	83.33	63.09	
		PFP3	H	5.36	4.26	4.11	4.14	3.81	
			C	109.16	81.40	77.71	83.33	62.84	
		PFP4	H	5.35	4.24	4.12	4.13	3.82	
			C	109.63	81.47	76.69	83.07	62.80	
**B**	*β*-_D_-Xyl*p*	PFP2	H	4.67	3.38	3.49	3.67	3.79	
			C	105.24	74.91	76.26	70.93	66.26	
		PFP3	H	4.66	3.38	3.45	3.66	3.78	
			C	104.90	73.28	76.85	71.74	66.02	
		PFP4	H	4.64	3.38	3.50	3.66	3.76	
			C	105.27	74.70	76.20	72.60	66.12	
**C**	*β*-_D_-Gal*p*	PFP2	H	4.57	3.55	3.78	3.97	4.05	3.95
			C	103.73	72.18	72.89	70.79	74.73	62.35
		PFP3	H	4.56	3.53	3.77	4.00	4.05	3.95
			C	103.31	71.73	72.50	69.20	72.14	62.84
		PFP4	H	4.58	3.52	3.75	4.01	4.05	3.92
			C	103.14	72.11	72.80	69.40	73.10	62.80
**D**	→4)-*α*-_L_-Gal*p*Ac-(1→	PFP2	H	5.07	3.78	4.42	4.05	4.76	
			C	101.21	70.00	79.43	70.57	77.85	175.20
		PFP3	H	5.02	3.77	4.40	4.05	4.74	
			C	99.03	69.58	79.17	70.05	77.34	174.00
		PFP4	H	5.14	3.77	4.40	4.07	4.77	
			C	98.25	70.31	79.40	70.02	77.49	174.80
**E**	→4)-*α*-_L_-Man*p*-(1→	PFP2	H	5.33	4.17	3.67	3.84	3.75	4.04
			C	107.21	69.48	75.69	80.87	71.33	73.75
		PFP3	H	5.32	4.17	3.67	3.84	3.75	4.00
			C	106.34	67.90	75.43	80.13	71.50	71.60
		PFP4	H	5.30	4.17	3.67	3.83	3.75	3.99
			C	107.14	68.90	75.78	80.89	71.61	70.39
**F**	→3)-*α*-_L_-Gal*p*-(1→	PFP2	H	5.14	3.78	4.07	3.94	3.49	3.63
			C	99.55	70.00	83.61	61.33	73.32	60.00
		PFP3	H	5.22	3.77	4.05	3.93	3.48	3.60
			C	100.96	69.58	83.91	60.88	73.05	59.55
		PFP4	H	5.21	3.77	4.05	3.93	3.49	3.59
			C	101.54	70.31	82.07	61.12	73.49	59.84
**G**	→3, 4)-*β*-_D_-Gal*p*Ac-(1→	PFP2	H	4.83	3.75	4.35	4.51	5.19	
			C	103.99	71.33	68.61	79.92	70.88	174.11
		PFP3	H	4.92	3.75	4.34	4.49	5.18	
			C	101.22	71.50	68.22	79.35	70.48	170.70
		PFP4	H	4.97	3.75	4.34	4.47	5.17	
			C	102.64	71.61	68.60	81.77	70.81	173.20
**H**	→3, 6)-*β*-_D_-Gal*p*-(1→	PFP2	H	4.90	3.67	3.73	4.17	3.85	3.89
			C	100.91	70.93	79.29	69.48	76.28	71.98
		PFP3	H	4.81	3.66	3.74	4.17	3.80	3.88
			C	103.58	71.74	79.95	69.48	76.42	71.96
		PFP4	H	4.82	3.66	3.74	4.17	3.81	3.88
			C	104.12	72.60	81.47	68.90	76.08	72.30
**I**	→6)-*α*-_L_-Glc*p*-(1→	PFP2	H	4.96	3.89	3.47	3.32	3.67	3.97
			C	104.01	71.98	75.13	68.55	75.69	70.79
		PFP4	H	4.94	3.88	3.45	3.32	3.67	4.01
			C	104.05	72.30	74.90	69.21	75.78	69.40
**J**	→6)-*β*-_D_-Gal*p*-(1→	PFP2	H	4.76	3.55	3.74	3.97	3.89	4.04
			C	104.35	75.921	76.55	70.79	71.98	73.75
		PFP4	H	4.74	3.62	3.76	4.01	3.88	3.99
			C	104.59	75.89	77.08	69.40	72.30	70.39
**K**	→2, 3)-*α*-_L_-Gal*p*-(1→	PFP2	H	5.07	4.07	3.98	3.75	3.79	3.81
			C	101.85	83.61	82.13	71.33	68.05	63.09
